# Early Life Stress and Risks for Opioid Misuse: Review of Data Supporting Neurobiological Underpinnings

**DOI:** 10.3390/jpm11040315

**Published:** 2021-04-19

**Authors:** Lynn M. Oswald, Kelly E. Dunn, David A. Seminowicz, Carla L. Storr

**Affiliations:** 1 Department of Family and Community Health, University of Maryland School of Nursing, Baltimore, MD 21201, USA; cstorr@umaryland.edu; 2 Behavioral Pharmacology Research Unit, Department of Psychiatry and Behavioral Sciences, Johns Hopkins University School of Medicine, Baltimore, MD 21230, USA; kdunn9@jhmi.edu; 3 Department of Neural and Pain Sciences, School of Dentistry, University of Maryland, Baltimore, MD 21201, USA; dseminowicz@umaryland.edu; 4 Center to Advance Chronic Pain Research, University of Maryland, Baltimore, MD 21201, USA

**Keywords:** early life stress, opioid use disorder, opioid sensitivity, mesocorticolimbic, endogenous opioid, dopamine, emotion processing, reward processing

## Abstract

A robust body of research has shown that traumatic experiences occurring during critical developmental periods of childhood when neuronal plasticity is high increase risks for a spectrum of physical and mental health problems in adulthood, including substance use disorders. However, until recently, relatively few studies had specifically examined the relationships between early life stress (ELS) and opioid use disorder (OUD). Associations with opioid use initiation, injection drug use, overdose, and poor treatment outcome have now been demonstrated. In rodents, ELS has also been shown to increase the euphoric and decrease antinociceptive effects of opioids, but little is known about these processes in humans or about the neurobiological mechanisms that may underlie these relationships. This review aims to establish a theoretical model that highlights the mechanisms by which ELS may alter opioid sensitivity, thereby contributing to future risks for OUD. Alterations induced by ELS in mesocorticolimbic brain circuits, and endogenous opioid and dopamine neurotransmitter systems are described. The limited but provocative evidence linking these alterations with opioid sensitivity and risks for OUD is presented. Overall, the findings suggest that better understanding of these mechanisms holds promise for reducing vulnerability, improving prevention strategies, and prescribing guidelines for high-risk individuals.

## 1. Introduction

### 1.1. Prevalence of Early Life Stress (ELS)

In 2018, United States (US) Child Protective Service (CPS) agencies received an estimated 4.3 million referrals involving approximately 7.8 million children [1]. Approximately one-fifth of the children investigated were determined to be victims of maltreatment (abuse or neglect). The youngest and most vulnerable children (aged birth to one-year) have the highest rates of victimization at 26.7 per 1000. While these numbers are appalling, they probably represent only the tip of the iceberg as they do not include cases that go unreported or are unverified or cases that involve other forms of traumatic experiences. Findings from one of the largest and most diverse studies on adverse childhood experiences conducted to date revealed that 62% of the 248,934 non-institutionalized adults sampled across the US reported at least one traumatic experience during childhood and about a quarter reported at least three [2].

Adverse childhood experiences, also referred to as early life stress (ELS), may include events such as physical, sexual, or emotional abuse or neglect; exposure to domestic violence; family dysfunction; divorce; or death of a parent [3], which are outside the control of the child, have the potential to impair normal development, and may impair the child’s physical and/or psychological well-being [4]. The events often reflect either a threat involving harm to the physical integrity of the child or deprivation involving the absence of expected support, nurturance, or environmental enrichments [5]. Risk factors for maltreatment include caregivers with alcohol or drug problems, which are present in 12.3% and 30.7% of cases reported to the CPS, respectively. The rates of both of these risk factors have increased in the past several years, which is believed to be at least partially related to the increasing misuse of both prescription and non-prescription opioids [1].

### 1.2. Consequences of ELS

A robust body of research has now demonstrated that ELS exposure during early development when neuronal plasticity is high seems to have particularly damaging effects on an individual’s well-being. A history of severe or prolonged ELS is associated with increased likelihood of engaging in health risk behaviors, such as smoking, drug taking, and suicide attempts, as well as heightened risks for an array of emotional and physical health problems across their lifespan [6,7,8,9]. Indeed, findings of a growing number of studies, beginning with the landmark Adverse Childhood Experiences (ACE) study [10], suggest that there is a “dose–response” relationship between ELS and adult pathology, such that greater trauma is associated with a greater likelihood of such problems and with worse prognosis [11,12,13,14,15,16,17].

Epidemiological studies have identified positive associations between ELS and substance use disorders (SUDs) in both adolescents and adults [18,19,20]. While the majority of these findings have not been specific to opioid use disorder (OUD), the number of studies targeting opioids has grown in recent years. Consistent with the relationships observed between ELS and other types of SUDS, the findings show that ELS is associated with increased risks for opioid use initiation, injection drug use, overdose, use disorder, and poor treatment outcome [21,22,23,24,25,26,27]. Nevertheless, empirical studies elucidating the neurobiological mechanisms linking ELS with vulnerability for OUD are lacking.

### 1.3. Aim of this Review

**Figure 1:**
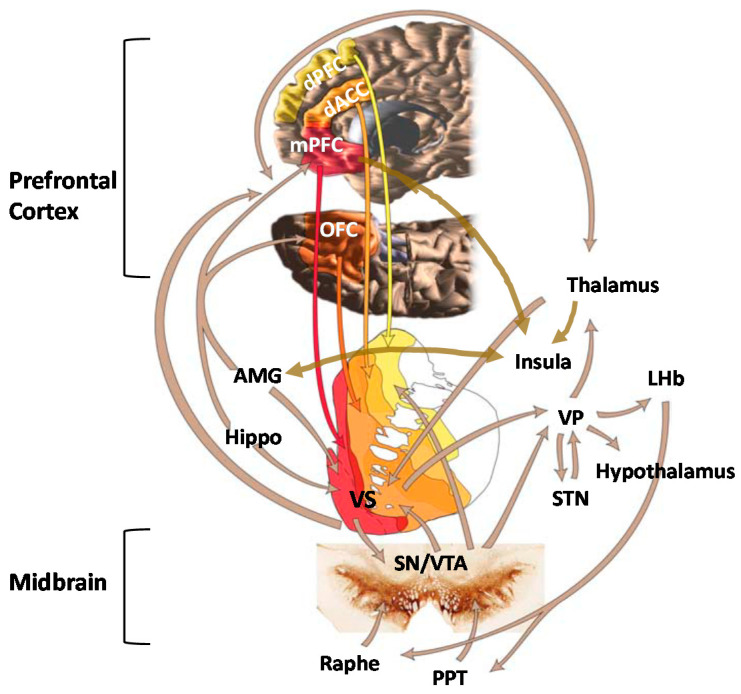
Key structures of the mesocorticolimbic system include the ventral tegmental area (VTA); ventral striatum (VS); hippocampus (Hippo); amygdala (AMG); thalamus; insula; hypothalamus; and cortical regions, including the dorsal anterior cingulate cortex (dACC), medial prefrontal cortex (mPFC), orbitofrontal cortex (OFC), and dorsal prefrontal cortex (dPFC). Additional structures shown on the figure are the ventral pallidum (VP), lateral habenula (LHb), subthalamic nucleus (STN), substantia nigra (SN), and pedunculopontine nucleus (PPT). Adapted from Haber and Knutson, 2010.

There is substantial and well-recognized variation in opioid sensitivity across individuals [28,29,30], which has been proposed to confer different levels of risk for opioid misuse and eventual OUD [31]. This review aims to establish a theoretical model (Figure 1) that summarizes the potential neurobiological mechanisms by which ELS alters opioid sensitivity, thereby contributing to future risks for OUD. This review first describes individual variation in risks for opioid misuse, the public health implications of opioid misuse/OUD, and the relationships between ELS and OUD. Next, the effects of ELS on mesocorticolimbic circuits that underlie emotion and reward processing and on the endogenous opioid and dopamine (DA) neurotransmitter circuits that play fundamental roles in these processes are discussed. Evidence linking these changes with individual differences in opioid sensitivity and risks for OUD is presented in each of the sections. The overarching hypothesis is that ELS-induced derangements in mesocorticolimbic brain regions lead to altered opioid sensitivity, which increases the abuse liability of these drugs and represents a preexisting vulnerability phenotype for OUD.

## 2. Individual Differences Confer Differential Risk for OUD

### 2.1. Opioid Sensitivity

Animal studies examining characteristics of drug intake repeatedly find that a subset of animals display a strong preference for opioids over other reinforcers and that preference is opioid-specific and does not represent a general sensitivity to reinforcing substances. For instance, rats given the opportunity to self-administer heroin versus other drug (e.g., cocaine) or non-drug (e.g., saccharin) reinforcers frequently develop an immediate preference for one but not all substances; this preference predicts future heroin-administration and development of “heroin addiction” [32,33,34,35,36]. Such individual differences in preference are also evident in nonhuman primates; one study found that nonhuman primates exposed to five different psychoactive substances (cocaine, remifentanil, methohexital, ethanol, and ketamine) developed strong individual preferences for a single drug class in a manner similar to what was observed in rats [37]. Although there are limited data to inform of this effect in humans, empirical support is growing. One human laboratory study found that a given individual generally experienced the same level of effect (positive or negative) when administered the opioids heroin and hydromorphone but that the level of effect differed markedly across individuals, suggesting that humans also display pronounced individual differences in risk for opioid misuse [38]. Moreover, retrospective human cohort studies have repeatedly found that patient self-reported experiences of their first opioid exposure varied widely across individuals and that the subset of participants who experienced an initial euphoric effect were more likely to develop OUD [39,40], suggesting that experiencing euphoria during the first opioid exposure was not a universal response but did signal a risk for opioid misuse [41].

### 2.2. Mechanisms Underlying Opioid Sensitivity

The mechanisms underlying variations in opioid effects have not yet been clearly elucidated but are hypothesized to include an array of pharmacokinetic, pharmacodynamic, and pharmacogenetic contributions [42]. For instance, rats that were tested with four different mu agonists displayed strong within-subject consistency but substantial dose variability (30- to 300-fold) across animals with regard to analgesia [43], suggesting a strong biological mechanism underlying differences in opioid sensitivity. An examination of opioid effects across different animal strains revealed that the opioid dose needed to produce equivalent levels of conditioned place preference (CPP, a measure of drug reward) was substantially and consistently higher among Wistar versus Sprague Dawley rats and corresponded to differential levels of DA release in the nucleus accumbens (NAc) [44]. Such a variability is also evident within the same animal strain; one study observed a continuum of responses in Sprague Dawley rats ranging from the 10% of animals showing a preference for morphine over food after 4 days to another 10% of the sample not developing a morphine preference even after 38 days of access [45]. A final study observed pronounced differences in the rate of heroin and oxycodone intake and preference across the four different sub-strains of the 129 mouse [46]. These strain-based differences suggest a pharmacogenetic contribution to individual differences; the most frequently explored gene in the context of opioid use has been OPRM1, which codes for the mu opioid receptor. OPRM1 appears to produce clinically meaningful differences in human opioid sensitivity [47,48,49,50] and to modulate human cortisol stress response [51], thus providing a putative pathway by which ELS may confer a unique risk for opioid misuse and OUD.

Stress has been independently associated with opioid sensitivity, with several studies observing evidence of greater OUD risk (e.g., increased opioid self-administration and drug reinstatement) [52,53,54] among animals exposed to stress from restraint [55], foot-shock [52,56], and intermittent swim [57] assays. Stressful stimuli have also been shown to produce a hyporesponsive state of the endogenous opioid system in animals [58], and corticosterone release following a stressful stimulus has been causally and independently linked to increased opioid self-administration [57]. Early life stress appears to be particularly destructive because it produces enduring conformational changes in the endogenous opioid system that impact drug use behavior [59,60]. The deleterious impact of ELS can be observed as far back as gestational exposure, wherein pups whose mothers were exposed to stress during gestation were more likely to acquire morphine CPP during their adolescence [61]. Converging evidence indicates that ELS in animals reduces opioid (particularly mu) receptor availability [62,63,64] and decreases downstream dopamine signaling [63,65] in a way that may bolster the reinforcing effects of opioids. Such an effect is also evident in behavioral tests where young animals exposed to stress display more CPP for µ- versus κ-opioid agonists [66]. Altogether, these data suggest that ELS may produce conformational changes in the opioid system that enhance opioid sensitivity, which may serve as the basis for the pronounced behavioral effects observed among adults.

## 3. Societal Impact of Opioid Misuse/Use Disorder

### 3.1. Epidemiological Findings

Not all persons who are exposed to opioids develop OUD, but those who do can experience devastating consequences. Opioids are considered essential medicines for acute and cancer pain, palliative care, and treatment of opioid dependence by the World Health Organization [67] and are used for medicinal purposes worldwide. Although the availability and consumption of opioid analgesics are considered inadequate to provide sufficient pain relief in some regions [68], in high-income countries, such as the US and Canada, increased prescribing and availability, aggressive promotion, and under-regulation of these drugs from the 1990s to around 2011 has led to a serious national crisis and spiraling needs for healthcare services. In 2018, 57.8 million people globally were estimated to have used opioids in the past year, with almost half of them misusing pharmaceutical opioids [69]. In fact, prescription pain reliever misuse in the US is second only to marijuana as the first illicit substance that people try, with approximately 4400 new initiates each day [70].

Examination of trends in opioid analgesic abuse from 2002 to 2011 showed that approximately 75% of US heroin users reported being introduced to opioids through prescription drug use [71,72]. However, despite evidence that misuse of prescription pain relievers has declined (from 4.7% in 2015 to 3.5% in 2019), heroin use has remained relatively stable over the past decade. It is estimated that 1.6 million people in the US suffered from OUD in 2019 [70]. These problems are accompanied by school dropout, unemployment, poor quality of life, cooccurring psychiatric disorders, and problems with the criminal justice system [73], which represent not only tremendous burdens to individuals and families but also a US “economic burden” of roughly $78.5 billion a year [74]. Two out of three drug overdose deaths in the US in 2018 involved an opioid, which can be attributed to the wider availability of high potency opioid analgesics and synthetic opioids since about 2013 [73,75,76].

### 3.2. Risk Factors

Risk factors for opioid misuse and OUD include both genetic and environmental determinants. Findings of twin studies suggest that genetic variance explains approximately half of the liability for OUD, although some of this variance is likely a genetic disposition to drug use disorders in general [77,78,79]. However, environmental factors also play a prominent role, including drug availability, peer pressure, other substance use, adverse childhood experiences, family history of alcohol and drug use disorder, and other comorbid mental health problems [71,73,80,81,82,83,84]. Predictors of prolonged opioid use in patients with musculoskeletal problems include past or current substance use problems, higher initially prescribed doses, mood disorders, and depression [71,85,86]. Among chronic pain patients, predictors of misuse include anxiety, anger, pain intensity, and depression [87,88,89] as well as measures of distress intolerance [90], pain catastrophizing [91], and difficulties in emotion regulation [92].

## 4. Evidence of ELS and OUD Associations

### 4.1. ELS Is Highly Prevalent among Persons with OUD

An extensive body of research has shown that ELS exposure profoundly increases risks for the development of alcohol, cocaine, marijuana, and nicotine use disorders [18,93,94,95,96,97], but only recently has it become evident that childhood adversity is also common among individuals with OUD [22,98,99,100,101]. A recent meta-analysis found that 41% of women and 16% of men with OUD reported a history sexual abuse and that 38–42% reported other types of mistreatment before the age of 18 [100]. Early life stress has also been linked to higher rates of relapse, suicidal ideation, overdose, and a more rapid transition from misuse to OUD in these individuals [21,22,102,103].

Data from the National Longitudinal Study of Adolescent to Adult Health study (*n* = 12,288) revealed a dose–response relationship between ELS and adulthood prescription pain reliever misuse that increased in strength from young to middle adulthood [104]. There is also evidence that the odds of prescription opioid/pain reliever misuse increase as a function of the number of ELS events experienced [26,104,105,106] and that other behaviors, such as age of first opioid use and injection drug use, are also associated with the number of early life stressors endorsed [25,104,106]. Although the aggregate number of events seems to have a cumulative effect [22,107], the type of early life stressor may also impact the outcomes. For example, in one study, neglect, emotional abuse, and parental incarceration were associated with 25–55% increased odds of prescription pain reliever misuse in young adults while sexual abuse and witnessing violence were associated with nearly three and five times the odds of injection drug use [104]. Chronicity, severity, and developmental timing of these experiences are also differentially related to drug use outcomes [18].

### 4.2. Persons with ELS and/or OUD Exhibit Similar Pathologies

Early life stress is associated with deficits in emotion regulation and neuroendocrine responses to stress that persist into adulthood and are associated with the development of several forms of psychopathology [5,108,109,110,111,112,113,114,115,116,117,118]. Recently, it has been suggested that such deficits may be intermediaries in the pathway that links ELS with the development of OUD [90,103,119,120,121]. For instance, emotional responses to neutral and unpleasant stimuli were positively associated with childhood neglect and severity of addiction in heroin users, suggesting that ELS impairs the ability to modulate emotions and to cope with stress. These deficits then give rise to a range of maladaptive behaviors that can predispose to OUD [122]. Similar findings were reported by Ghorbani et al. [123], who showed that ELS was indirectly related to heroin craving via a limited ability to regulate emotions. Internalizing and externalizing symptoms have been shown to partially mediate the association between ELS and prescription opioid misuse [24,106]. These findings suggest that self-medication may play a role in generating and maintaining both recreational and prescription opioid misuse in ELS-exposed individuals [124,125]. In addition to their euphoric and analgesic effects, opioids relieve stress through their inhibitory actions on the hypothalamic-pituitary-adrenal (HPA) axis [120,126,127], which can be highly negatively reinforcing [128] to individuals with such affective vulnerabilities.

## 5. Role of Mesocorticolimbic Emotion Processing Circuits in ELS and OUD

### 5.1. Effects of ELS on Emotion Processing Circuits

Mesocorticolimbic brain circuits include several cortical and subcortical structures that are integrally involved in stress regulation and emotion and reward processing [129] (Figure 1). Two regions that seem to be particularly impacted by ELS are the amygdala (AMG) and medial prefrontal cortex (mPFC) [130,131,132,133]. These structures have extensive bidirectional connections and normally work together to integrate the expression of fundamental aspects of emotional learning, memory, and behavior as well as play prominent roles in stress responsivity [134,135,136]. As maturation of these regions occurs throughout juvenile and adolescent periods, perturbations that occur during childhood can alter their normal neurodevelopmental trajectories, which may negatively impact psychological function later in life [135]. Alterations in AMG volume [137,138] and task-related hyper-responsivity [133,139,140,141,142,143] have been observed in both adults and children with a history of ELS. Oshri et al. [144] showed that ELS-related changes in AMG volume were associated with increased anxiety, depressive symptoms, and alcohol use. Heightened threat-related AMG reactivity has been shown to predict internalizing symptoms [145] and risks for alcohol use disorder [146,147]. In contrast, ELS has been associated with reduced mPFC volume [148,149] and lower mPFC activation during both resting state and cognitive tasks [150,151,152].

Normally, the mPFC, particularly the ventral mPFC, modulates fear behavior by providing top-down control of AMG and HPA-axis responses to stress [153,154]. Dysfunction in AMG-mPFC connectivity has been implicated in several psychiatric disorders, including depression and schizophrenia [135], and there is evidence that top-down control may be altered in individuals with a history of ELS. While valence (positive or negative) and regional specificity vary somewhat across studies [136], atypical patterns of connectivity have been observed between the AMG and PFC in both youth and adults with a history of ELS. Findings in children and adolescents include evidence of weakened left AMG–anterior cingulate cortex (ACC) resting state functional connectivity (rs-FC), which was associated with higher levels of current anxiety in one study and mediated the relationship between ELS and internalizing symptoms in another [155,156,157]. Gee et al. [140] reported precocious maturation of the mPFC–AMG pathway in previously institutionalized youth, which reflected a developmental shift from positive to negative coupling and seemed to confer some degree of reduced anxiety. These somewhat counterintuitive findings suggested that accelerated maturation of these connections may serve to facilitate coping with environmental insults in the short term but lead to less efficient stress regulation and increased vulnerability for psychopathology in adulthood [135]. Associations between AMG–PFC connectivity and cortisol levels have also been reported in humans [158] and nonhuman primates [159], suggesting that ELS-related changes in neuroendocrine function contribute to the neural deficits and problems with emotion regulation that are observed in these individuals in later life.

Similar ELS-related findings have been reported in adults, including weakened AMG–pregenual ACC rs-FC, which predicted elevated state anxiety [160], and reduced AMG–ventral mPFC rs-FC, which predicted increased levels of pro-inflammatory cytokines [161]. However, inconsistent findings have also been reported, which may be related to variations in network configurations between resting state and task-related co-activations, task-specific engagement, or other methodological differences among studies. In contrast to the findings during resting state scans, Jedd et al. [162] reported increased AMG–PFC during an emotion processing task in ELS-exposed adults. Kaiser et al. [163] found that ELS was associated with greater negative AMG–dorsolateral PFC rs-FC, which mediated the relationship between ELS severity and blunted cortisol response to acute stress, and elevated dynamic AMG–rostral ACC rs-FC, which was associated with reduced negative mood following a social evaluation stress challenge. These findings suggested that ELS may be associated with both maladaptive and compensatory changes in mesocorticolimbic circuits. Though most studies in this area of research are cross-sectional and preclude determinations of causality, overall, these findings suggest that neuroplastic aberrations incurred as a result of ELS may persist decades later into adulthood, leading to alterations in physiological and emotional responses to stress.

There has been growing evidence that the effects of ELS on mesocorticolimbic brain structures may be partially related to repeated or chronically high levels of corticotropin-releasing factor (CRF) and glucocorticoids (GCs) [164,165,166,167,168,169] that occur during early stages of ELS [170,171,172]. Although other mechanisms may be involved, composite findings from several lines of research indicate that epigenetic processes involving HPA-axis regulation and GC signaling underlie many of these effects [173,174]. Glucocorticoid receptors (GR) are densely located throughout the brain, including stress-sensitive regions such as the mPFC, AMG, hippocampus, NAc, and hypothalamus [175,176]. Regional differences in methylation and both increases and decreases in brain GR expression have been observed in rodents and monkeys exposed to ELS [177,178,179,180]. Findings of one postmortem study showed decreased levels of GR mRNA in the hippocampus of suicide victims with a history of ELS compared to those without this history [181]. Greater GC receptor methylation has also been found in the peripheral cells of ELS-exposed humans [182], which was recently shown to moderate associations between ELS and cortisol stress reactivity [183].

### 5.2. Emotion Processing Circuit Interface between ELS and OUD

Although OUD is underrepresented in the neuroimaging literature on addiction compared to other SUDs [184], findings from a limited number of human fMRI studies have implicated brain circuits involved in stress and emotion regulation in the perpetuation of this disorder. For example, the AMG has been shown to be activated in response to heroin drug cues [185,186,187] and plays a central role in the generation of cue-elicited craving in persons with OUD [188]. Schmidt et al. [189] found that patients with OUD displayed higher left AMG response to fearful faces than healthy controls during acute withdrawal, which correlated with levels of state anxiety, ACTH, and cortisol levels in all subjects and with heroin craving in patients. However, AMG connectivity was reduced to levels that did not differ from those of healthy controls after acute heroin maintenance treatment, suggesting that the results were driven by the drug-related attenuation of stress hormone release [189]. Alterations have also been found in mPFC activation and connectivity in abstinent, currently using, and methadone-maintained persons with OUD during resting state and cue reactivity, inhibitory control, and emotion processing tasks [190,191,192,193]. Administration of the opioid antagonist naltrexone has been shown to decrease AMG and to increase PFC responses to drug cues in abstinent heroin users, which suggests that naltrexone’s clinical effects may result in part from its ability to increase the capacity for conscious self-regulation [186]. Wang et al. [194] further showed that greater pretreatment mPFC response to heroin-related cues predicted greater adherence to naltrexone in detoxified heroin-dependent individuals, demonstrating that lower mPFC activity may contribute to increased craving, negative affect, and reduced treatment compliance. In general, the imaging data suggest that OUD is associated with reduced PFC monitoring, weak inhibitory controls, dysfunctional stress responses, problems with emotion regulation, and heightened negative affective states.

Nevertheless, in spite of the commonalities in behavioral and neural deficits that have been observed between ELS-exposed individuals and persons with OUD, to our knowledge, relationships between ELS-induced changes in brain function and opioid misuse have never been examined. Findings from several lines of research support speculations that ELS-related changes in circuits that underlie stress and emotion processing may represent an underlying opioid vulnerability pathway. For instance, impairments in AMG connectivity have been shown to mediate associations between ELS and internalizing symptoms in adolescents [155] while internalizing and externalizing symptoms partially mediate associations between ELS and opioid misuse [24,106]. These findings suggest that ELS may lead to changes in brain function that impair the ability to modulate emotions and to cope with stress, which may then lead to a range of maladaptive behaviors that can predispose to opioid misuse. Poor coping and emotion regulation profiles have previously been shown to predict initiation of opioids at an earlier age, past 90-day heroin use, increased probability of injecting a drug, and less likelihood of heroin abstinence after treatment, which may all be related to a need for self-medication [195].

It is important to note that these findings do not specifically explain the preference for opioids in these individuals as poor coping and emotion regulation have also been associated with misuse of other substances [195,196]. However, findings of a growing body of research suggest that ELS-related changes in emotion processing regions may lead to altered opioid sensitivity. Prefrontal and NAc activation are partially mediated by the endogenous opioid system [31,197], which is altered in ELS-exposed individuals. In one study, opioid antagonist naltrexone modulated activation of the mPFC during negative emotional processing as a function of ELS in both healthy controls and individuals with alcohol, cocaine, and/or opioid use disorders [198]. Specifically, the greater the severity of abuse, the greater the sensitivity of the mPFC to the effects of naltrexone, suggesting that ELS-related changes in mPFC function may underlie individual differences in responsiveness to the drug. Persons with AUD/SUD also reported higher levels of depression, anxiety, and stress sensitivity than the control group, which was somewhat ameliorated by naltrexone. These data are consistent with preclinical evidence showing that naltrexone reduces ethanol consumption in rats that experienced prolonged separation but not short absences of the dam [199]. Collectively, the data support hypotheses that ELS-induced derangements in endogenous opioid function in stress and emotion processing circuits may underlie part of the variability detected in opioid sensitivity across individuals. However, significant gaps still remain in our understanding of how ELS-induced changes in these circuits influence the course of opioid addiction and why alterations in function are associated with maladaptive behaviors in some individuals but not others.

## 6. Role of Mesocorticolimbic Reward Processing Circuits in ELS and OUD

### 6.1. Effects of ELS on Reward Processing Circuits

Reward functions underlying addiction are generally thought to involve the ventral tegmental area (VTA) and mesolimbic DA neurons that project from the midbrain VTA to the ventral striatum (VS) or, more specifically, the NAc, which is considered the center of reward learning. It is well-established that ELS increases feelings of dysphoria and anhedonia in humans and leads to an attenuation of behavioral responses to primary and conditioned rewards in animals [200,201,202,203,204,205]. Several investigators have hypothesized that these outcomes may be the result of ELS-induced alterations in NAc function. Goff et al. [206] reported hypoactivation of the NAc during an emotional faces task in adolescents with a history of ELS, which was associated with higher levels of depression. Between-subject comparisons of children ages 5–10 yrs. and adolescents 11–15 yrs. old further indicated that the ELS group failed to show the developmentally typical rise in NAc reactivity shown by the comparison group. The findings suggested that ELS impacts the development of the VS, resulting in hypoactivity, which leads to dysfunctional reward and motivational processing. Other investigators have shown similar findings consistent with notions of reduced activation of striatal structures and dampened behavioral responses to reward in ELS-exposed individuals [203,207,208]. Given prior evidence that blunted reward responsivity may be a marker of motivational mechanisms underlying addiction vulnerability [209,210,211,212,213], these findings suggest that alterations in vs. function may be one mechanism that underlies increased susceptibility for addiction in ELS-exposed individuals.

Several cross-sectional fMRI studies have examined associations between ELS-induced changes in reward circuitry and subjective measures of reward [214,215,216]. For example, Dillon et al. [203] found that ELS-exposed young adults reported elevated depressive and anhedonic symptoms, rated reward cues less positively, and exhibited decreased anticipatory reward activity in left basal ganglia regions relative to controls during completion of a monetary reward task. Corral-Frias et al. [217] showed that blunted vs. reactivity to reward was associated with increased anhedonic symptoms, which indirectly predicted other depressive symptoms and problematic alcohol use in ELS-exposed young adults. Marusak et al. [218] examined blood-oxygen-level-dependent (BOLD) responses to an emotional conflict task, showing that greater conflict-related AMG reactivity was also associated with diminished trait reward sensitivity in adolescents with a history of ELS. Overall, these findings are consistent with behavioral evidence from preclinical studies showing that ELS impairs motivation to work for rewards in animals, suggesting a downregulation of reward functions [219,220,221,222].

Longitudinal studies are somewhat rare in this area of investigation. However, Birn and colleagues [223] found that young adults who experienced a high level of stress as children showed deficits in decision-making during a reward processing task and reported more real-life risk-taking behaviors than individuals without this history. They also displayed alterations in reward processing regions, including the middle temporal gyrus, precuneus, putamen, insula, and left inferior frontal gyrus, some of which mediated relationships between ELS and reward processing or self-reported risk-taking behavior. Casement et al. [224] showed that cumulative life stress from ages 15–18 years was associated with decreased mPFC response during both anticipation and receipt of monetary reward at age 20 in adult males. The blunted mPFC response to reward predicted greater symptoms of alcohol dependence and mediated the relationship between life stress and alcohol use. Boecker et al. [225] found that ELS assessed at 3 months after birth and between the ages of 2 and 15 years was associated with hyporesponsiveness in reward circuits during reward anticipation (i.e., VS, putamen, and thalamus) and hyperresponsiveness during reward delivery (i.e., insula, pallidum, substantia nigra, and right posterior hippocampus) in healthy young adults who had been followed prospectively over 25 years.

To date, only a small number of studies have examined the influence of ELS on functional connectivity in reward processing regions. Fareri et al. [226] found that resting state coupling between the VS and mPFC was stronger in previously institutionalized youth than in youth who were raised by their biological parents; the fMRI findings mediated differences in social problems between the two groups. Blunted maturation of VTA–mPFC resting state connections [227] and increased connectivity of the insula to salience network seed regions have also been observed in trauma-exposed youth, which was associated with diminished reward sensitivity [216]. Hanson et al. [228] found elevated VS–mPFC functional connectivity during a monetary reward task in college-age adults with a history of both ELS and higher levels of recent life stress, which suggested that the deficits observed in youth persist into young adulthood. Although further prospective research is needed, collectively, the findings suggest that blunted VS activity and elevated functional connectivity in reward processing regions may represent neurobiological markers that serve as indicators of diathesis for psychological dysfunction in adults with a history of ELS. Overall, the findings are consistent with those of preclinical research showing broad changes in connectivity of the limbic and reward networks of adult rats exposed to ELS [229] as well as deficits in reward responsiveness and approach motivation [220].

### 6.2. Reward Processing Circuit Interface between ELS and OUD

In recent years, fMRI has been used to evaluate abnormalities in activation and functional connectivity of reward regions in abstinent and currently using heroin-dependent individuals using a variety of paradigms [184,230]. In general, brain activation has been shown to be upregulated in the reward and salience network brain regions of persons with OUD in response to drug-related cues [231]. Dysfunctional connectivity has also been reported using a variety of methods, including at rest, in response to heroin-related cues, and while performing decision-making or response inhibition tasks in regions that include the mPFC, orbitofrontal cortex (OFC), dorsolateral PFC, ACC, posterior cingulate cortex (PCC), and NAc [191,192,232,233,234,235]. In one study, rs-FC between regions involved in reward and motivation (e.g., VS-ACC and VS-OFC) was increased and connectivity between regions involved in cognitive control (e.g., PFC-ACC) was decreased in chronic heroin users, most of whom were being treated with methadone [236]. Functional connections between the AMG and mPFC have also been shown to be critical for the processing of opioid rewards [237,238,239]. Engagement of the reward networks is associated with craving, addiction severity, duration of use, and/or relapse in persons with OUD [191,240,241].

Preclinical studies have demonstrated that chronic opioid use produces abnormalities in mesocorticolimbic reward circuits that contribute to opioid misuse and OUD [191,239,242,243,244,245,246]. However, genetic and environmental factors, such as stress, may also lead to functional deficits in these circuits that underlie increased susceptibility for drug misuse and dysregulated responses to opioids. As previously described, ELS-related derangements in reactivity and connectivity of reward circuits have been associated with problematic alcohol use and risks for alcohol use disorder as well as well-established intermediate phenotypes for SUDs (e.g., anhedonia, deficits in decision-making, and problems with reward-based learning). However, virtually nothing is known about how ELS-induced alterations in reward circuits influence opioid sensitivity or about the role that such alterations play in the transition from opioid use to misuse. Similarities between ELS-induced alterations in connectivity and those observed in persons with OUD suggest that ELS-related derangements may predate and increase vulnerability for this disorder. This relationship is also suggested by evidence that ELS-induces changes in DA and endogenous opioid neurotransmission, which mediate the reward functions of these regions [247,248]. However, further research is needed to test these hypotheses.

## 7. Role of Endogenous Opioid Neurotransmitter System in ELS and OUD

### 7.1. Effects of ELS on Endogenous Opioid Function

**Figure 2:**
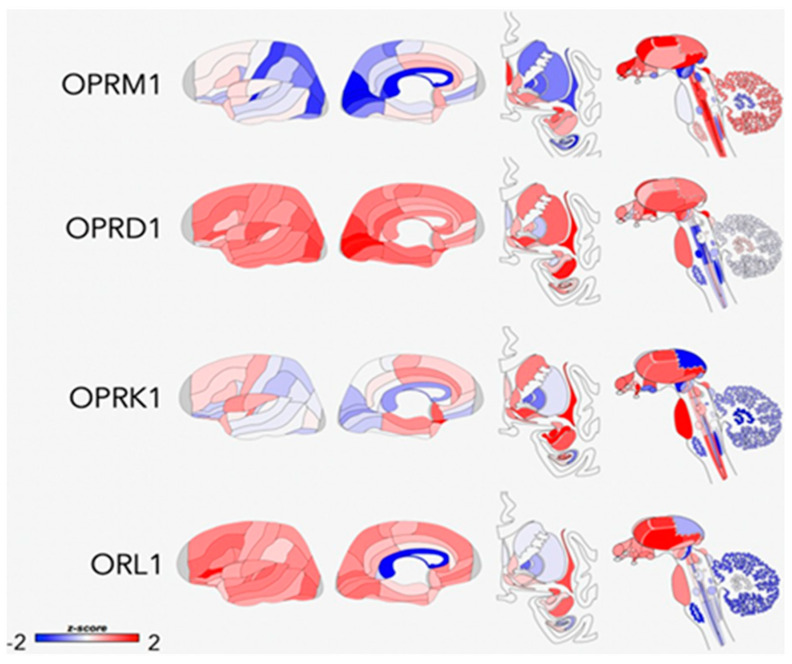
Areas of opioid receptor gene expression (*μ* = OPRM1, *δ* = OPRD1, *κ* = OPRK1, and NOP = ORL1) in the human brain (donor: H0351.1015, 55 yrs, male, and white or Caucasian). The cortical gene expression patterns are displayed on an inflated cortical surface (outer and inner surfaces of the left hemisphere). Subcortical structures of the brain are represented from the frontal view, and subcortical as well as brainstem structures are shown in the side view. The color bar displays expression values using z-score normalization. Reproduced from Peciña et al., 2019. Creative Commons license: http://creativecommons.org/licenses/by/4.0/, accessed on 14 April 2021.

Opioid peptides and receptors are widely distributed throughout the central nervous system and are thought to modulate many aspects of human behavior, including reward, affective states, pain responses, and other physiological functions [249] (Figure 2). Considerable variability in responses to opioid drugs has been noted in the general population, which are associated with differences in therapeutic response to treatment as well as risks for drug misuse [41,47,250,251]. Such differences may be due to both heritable factors and environmental influences that interact with the genome through epigenetic or transcription mechanisms to produce long-term alterations in the endogenous opioid system [252]. Endogenous opioid peptides are found throughout the peripheral and central nervous systems, where they play a role in many different types of functions, including nociception and analgesia, stress responses, physiological functions, social behavior, mood, and reinforcement [127,253,254,255,256,257]. Normally, this system is activated by acute stress, leading to the release of endogenous opioids at multiple sites in the brain. The release of opioids generally serves to attenuate stress responses by actions that include modulating the release of CRF, which returns the systems to pre-perturbation levels. However, repetitive stress exposure (which is characteristic of ELS) leads to an imbalance between CRF and opioids such that opioid inhibitory tone is favored [258]. Although the relationships require further testing, there is some evidence that chronically high tonic levels of endogenous opioids may trigger downregulation or reduced affinity of µ-opioid receptors (MOR) and lower phasic release of opioid peptides, resulting in hypoactivity of this system [259,260].

Conformational changes that have been specifically associated with ELS in preclinical studies include changes in opioid peptide levels [63,261], kappa receptor signaling [262], and variations in mu- and kappa-receptor (KOR) gene expression [62,64,65,263,264] in brain regions that include the hypothalamus, PFC, periaqueductal gray (PAG), AMG, NAc, rostral ventromedial medulla, and lateral habenula. Nylander and Roman [251] concluded that the most pronounced effect of ELS on opioid peptides is on Met-inkephalinArg6Phe7 (MEAP) levels, which are reduced in ELS-exposed animals. Rats with lower MEAP levels exhibit altered risk-taking behavior and a propensity for high ethanol intake, consistent with theories that an inherent opioid deficiency leads to increased susceptibility for addiction. Given the large number of physiological functions that are regulated by the endogenous opioid system, it is reasonable to speculate that hypofunction of this system could also lead to dysregulation of stress responses, altered pain-processing, and an array of stress-related disorders.

The first direct evidence of ELS effects on opioid neurotransmission in humans was reported by Lutz et al. [92], who conducted a postmortem study showing that ELS was associated with the downregulation of kappa receptors in the anterior insula of both depressed individuals who died by suicide and controls who died suddenly from accidental causes. Cortisol response to naltrexone has also been found to be blunted in high vs. low ELS women, which similarly linked ELS with downregulation of endogenous opioid activity [265]. The authors posited that these effects may reflect an adaptation of the central opioid system that shapes how a person responds to motivationally significant stimuli. The findings of a recent study by Garland et al. [266] are consistent with these notions, showing that ELS was associated with blunted heart rate variability (HRV) and increased cue-elicited drug craving during a task involving negative emotions in female opioid-treated chronic pain patients. In theory, the reduced capacity to respond to negative emotional stimuli could be the result of reduced opioid function. However, opioid function was not specifically examined and the cross-sectional nature of the study makes it impossible to know whether the deficits predated or were a result of chronic opioid use. It is also unclear whether the findings would be replicated in an opioid-naïve sample without prior history of chronic pain. Nevertheless, in spite of the limitations, aggregate findings from this small body of human studies are consistent with the preponderance of preclinical evidence suggesting that ELS leads to a deficiency in opioid neurotransmission.

### 7.2. Interface between ELS and OUD via Endogenous Opioid Function

Much of the current research on the neurobiology of OUD is focused on gaining a better understanding of the molecular and cellular aspects of opioid receptor function that contribute to vulnerability for this disorder [267] or on characterizing how genetic influences on receptor function translate to abuse liability and treatment outcomes [268,269,270]. Although this research holds promise for explaining some of the variability in clinical responses to opioids, the evidence that heritability estimates for OUD are only about 23–54% [271] suggests that there is also a need for better understanding of the effects of environmental factors that help to shape the behavioral and molecular profiles of individuals with this disorder.

Recent preclinical findings have provided initial evidence that ELS-induced changes in endogenous opioid function may alter opioid agonist and antagonist sensitivity. In one study, ELS-exposed rats showed greater place preference for the µ-agonist morphine but lesser aversion to the k-receptor agonist spiradoline [66], suggesting that ELS may enhance opioid abuse vulnerability by both increasing reward sensitivity and by decreasing the aversive effects at k-receptors [272]. Vazquez et al. [273] showed that maternal deprivation in rat pups was associated with hypersensitivity to the reinforcing effects of morphine and development of morphine dependence in adulthood, which was likely a result of basal hypoactivity of the nucleus accumbens (NAc) enkephalinergic system. Nakamoto et al. [64] found that ELS-exposed mice displayed decreased µ- and k-opioid receptor messenger mRNA expression in the PAG and increased k-opioid receptor expression in the AMG. A lack of morphine antinociception was observed in stressed mice in adulthood but not immediately after ELS exposure. Bruehl et al. [41] recently extended these findings to humans, showing that endogenous opioid function assessed by naloxone administration was inversely associated with euphoric effects of a single dose of 0.09 mg/kg morphine sulfate in patients with low back pain. According to reinforcement theory [274], either hypersensitivity to the reinforcing effects or diminished sensitivity to the antinociceptive effects of opioids could lead to misuse of these drugs, through positive or negative reinforcement, respectively. There is also evidence that ELS-induced changes in endogenous opioid function may lead to dysfunctions in DA neurotransmission in rats [63,275]. In one study, ELS increased KOR-mediated inhibition of baseline and stimulated DA release, which contributed to a hypodopaminergic state and escalated ethanol intake. Taken together, these findings provide support for hypotheses that ELS-induced conformational changes in the endogenous opioid system [58,273] may alter the effects of opioid drugs in ways that increase risks for their misuse [59,276,277,278].

Although not specific to OUD, the findings from human positron-emission tomography (PET) imaging studies have found associations between endogenous opioid function, and subjective and behavioral responses to both drugs of abuse and pain. For example, k-opioid receptor availability is associated with stress-induced cocaine self-administration in subjects with cocaine-use disorder [279], altered vs. binding potential for the µ-receptor agonist [^11^C]carfentanil is associated with alcohol craving and relapse risk in abstinent alcoholics [280,281,282], the µ-receptor binding potential correlates with nicotine dependence and reward in smokers [283], and alterations in endogenous opioids and µ-receptors in patients with chronic nonspecific back pain are associated with both sensory and affective elements of the pain experience [284]. To date, we are not aware of any neuroimaging studies that have evaluated the effects of ELS on opioid neurotransmission in humans or any controlled human laboratory studies that have examined ELS contribution to human opioid sensitivity. Better understanding of the role that individual differences in sensitivity play as mediators in the transition from prescription or recreational opioid use to opioid misuse and risky-drug related behaviors may help to inform the development of more effective interventions for ELS-exposed individuals.

## 8. Role of Dopamine Neurotransmitter System in ELS and OUD

### 8.1. Effects of ELS on Dopamine Function

Another neurotransmitter system that plays a fundamental role in stress responses and emotional-motivational activation of reward seeking is the midbrain DA system [285,286,287,288]. The DA mesolimbic pathway projects from the VTA to the VS, AMG, and hippocampus, and the mesocortical pathway projects from the VTA to cortical regions such as the ACC, OFC, mPFC, and insula. Considerable preclinical evidence has emerged showing that ELS may lead to profound and long-term derangements in brain DA neurotransmission. Abnormalities that have been observed include but are not limited to altered D1, D2, and D3 receptor mRNA expression; decreased density of DA transporters; increased DA metabolites in the striatum and/or NAc [289,290,291,292,293,294]; and reduced rates of DA clearance in the mPFC [295]. Both enhanced [290,296,297] and blunted [290,296,297,298] striatal DA responses to stress have been observed in adult rodents exposed to ELS. It has been suggested that, in general, ELS induces a hypodopaminergic state with an associated enhancement of DA system responses to salient stimuli [63]. There is also a general consensus that the effects of ELS on DA neurotransmission has broad-based clinical implications for the development of psychopathological conditions, such as schizophrenia and addiction, which are known to be associated with malfunctions in DA neurotransmission [96,299,300,301]. It is hypothesized that these effects may be the result of excessive exposure to glucocorticoids during early life, which impacts the organization and epigenetic control of midbrain DA systems [302,303,304,305].

Functional changes that occur in the DA system as a result of ELS may also be associated with altered neurochemical and behavioral responses to drug abuse. Early life stress may lead to enhanced DA and behavioral responses to psychostimulants and changes in drug consumption patterns that reflect greater vulnerability for drug abuse in later life in animals [275,290,297,298,305,306,307,308,309]. The first evidence that some of these findings may translate to humans was provided by findings of an [^11^C]raclopride positron emission tomography (PET) study conducted by Pruessner and colleagues [310], who found that persons who reported low maternal care had greater VS DA release in response to stress than individuals who reported high maternal care. Oswald et al. [311] extended this line of research by showing that ELS is also associated with enhanced VS DA responses to amphetamine in healthy young adults. The relationship between ELS and DA response was partially mediated by current levels of perceived stress, which suggested that ELS may not directly influence DA function in some individuals unless accompanied by elevated levels of psychological stress in adulthood.

### 8.2. Interface between ELS and OUD via Dopamine Function

It has been proposed that the reinforcing effects of opioid agonists are, at least, partially dependent on their actions on mesolimbic DA circuits [312,313,314,315]. Interactions between the DA and endogenous opioid systems are well-established within mesocorticolimbic brain circuits [316,317,318,319,320] and play a role in behavioral responses to opioids and in relief of pain in animals [321,322,323]. It is reasonable to posit that extensive ELS-related dysfunctions in DA circuits could, therefore, influence sensitivity to opioids and vulnerability for OUD. Blum and colleagues [210] hypothesized that addiction results from an underlying reward deficiency state characterized by hypodopaminergia. This condition may be innate or acquired and is clinically manifested as anhedonia, numbing, apathy, or decreased motivation for natural reinforcers [324,325]. According to the hypothesis, opioid/endorphin deficiency increases a person’s vulnerability for OUD by disrupting interactions between midbrain opioid and DA neurotransmitter systems [326]. This has been supported by evidence that ELS alters µ- and k-receptor mRNA levels [62,63,64] and decreases downstream DA signaling [63,65] in a way that may alter the reinforcing and antinociceptive effects of opioids. Dopamine-deficient mice display decreased sensitivity to the analgesic effects of morphine [313] and mice lacking DA D2 receptors fail to self-administer morphine [327], which also provides evidence that individual differences in DA function may contribute to differential opioid sensitivity.

## 9. Conclusions

**Figure 3:**
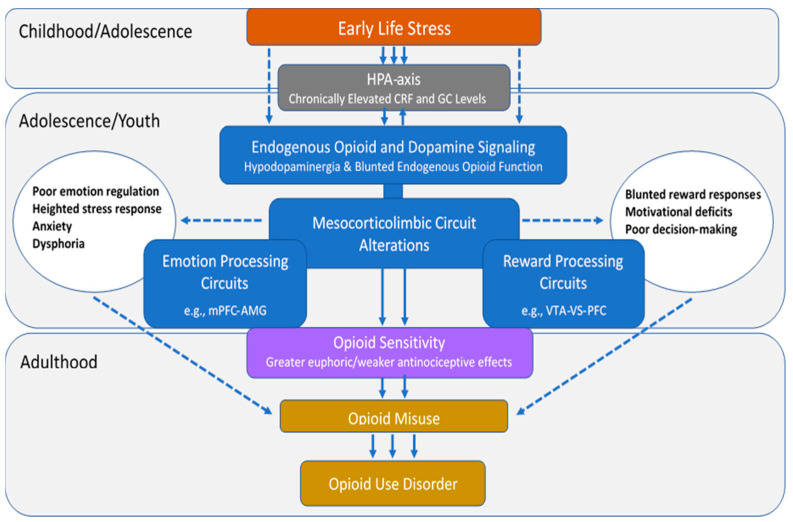
Putative pathway by which early life stress may alter opioid sensitivity and increase risks for opioid misuse/use disorder. The process begins with chronically elevated levels of corticotropin-releasing factor (CRF) and glucocorticoid (GC) levels that occur during the period of stress exposure, which have a long-term impact on endogenous opioid and dopamine neurotransmitter function, activation patterns, and connectivity in mesocorticolimbic emotion and reward processing regions. The neurobiological changes lead to increased euphoric and decreased antinociceptive responses to opioids and deficits in emotion regulation and reward processing in adulthood, which together may increase risks for recreational use or misuse of prescription opioids. The solid lines on the figure represent the direct pathway involving opioid sensitivity, and the dashed lines represent other mechanisms.

Epidemiological research has shown that ELS is highly prevalent in persons with OUD and is associated with opioid use initiation, injection drug use, overdose, and poor treatment outcome. However, despite evidence that ELS has a profound impact on mesocorticolimbic brain circuits implicated in OUD, the role that these alterations play in vulnerability for and severity of this disorder has yet to be elucidated. Figure 3 outlines a theoretical model based on the extant literature reviewed here, wherein ELS initiates a cascade of neurobiological changes that lead to altered opioid sensitivity and increased risks for OUD. This model is not meant to be exhaustive but to provide a foundation to support focused research in this area. The data suggest that ELS leads to chronically elevated CRF and GC levels in stress-sensitive mesocorticolimbic brain circuits during early exposure, which trigger changes in neurochemistry, activation patterns, and connectivity that persist into adulthood. The consequences include conformational changes in the endogenous opioid system that engender an endorphinergic deficiency and alterations in the DA neurotransmitter system that lead to hypodopaminergia. The presence of a vulnerability pathway involving opioid sensitivity is most strongly supported by preclinical evidence that ELS-induced changes in endogenous opioid function are associated with increased drug-seeking behavior and altered sensitivity to the reinforcing and antinociceptive effects of opioids and that DA-deficient mice exhibit decreased sensitivity to the analgesic effects of morphine, suggesting that DA function may also contribute to the ELS-exposed behavioral phenotype. Derangements in functional activation and connectivity in mesocorticolimbic circuits have been associated with emotion regulation and reward processing deficits in ELS-exposed humans and may reflect the underlying neurochemical imbalances in these regions. Such deficits may predispose an individual to a range of behaviors that independently increase opioid use and misuse. Once opioid use is initiated, ELS-induced neurochemical changes may be manifested as altered opioid sensitivity, which may facilitate the transition from use to misuse and ultimately OUD. Additional research that evaluates the effects of ELS exposure on opioid sensitivity and establishes the degree to which ELS-induced changes in endogenous opioid or DA neurotransmitter systems are present and/or contribute to these processes is warranted. This research could delineate key mechanisms underlying substantial individual variation in opioid risk and could lead to improved prevention strategies and prescribing guidelines for high-risk individuals.

## Data Availability

Not applicable.
